# Deletion of enzymes for de novo NAD ^+^ biosynthesis accelerated ovarian aging

**DOI:** 10.1111/acel.13904

**Published:** 2023-06-18

**Authors:** Qingling Yang, Hui Li, Huan Wang, Wenhui Chen, Xinxin Zeng, Xiaoyan Luo, Jianmin Xu, Yingpu Sun

**Affiliations:** ^1^ Center for Reproductive Medicine The First Affiliated Hospital of Zhengzhou University Zhengzhou China; ^2^ Henan Key Laboratory of Reproduction and Genetics The First Affiliated Hospital of Zhengzhou University Zhengzhou China; ^3^ Henan Provincial Obstetrical and Gynecological Diseases (Reproductive Medicine) Clinical Research Center The First Affiliated Hospital of Zhengzhou University Zhengzhou China

**Keywords:** de novo NAD^+^ synthesis pathway, mitochondrial function, ovarian aging, reactive oxygen species

## Abstract

Recent advances highlight the pivotal role of nicotinamide adenine dinucleotide (NAD^+^) in ovarian aging. However, the roles of de novo NAD^+^ biosynthesis on ovarian aging are still unknown. Here, we found that genetic ablation of *Ido1* (indoleamine‐2,3‐dioxygenase 1) or *Qprt* (Quinolinate phosphoribosyl transferase), two critical genes in de novo NAD^+^ biosynthesis, resulted in decreased ovarian NAD^+^ levels in middle‐aged mice, leading to subfertility, irregular estrous cycles, reduced ovarian reserve, and accelerated aging. Moreover, we observed impaired oocyte quality, characterized by increased reactive oxygen species and spindle anomalies, which ultimately led to reduced fertilization ability and impaired early embryonic development. A transcriptomic analysis of ovaries in both mutant and wild‐type mice revealed alterations in gene expression related to mitochondrial metabolism. Our findings were further supported by the observation of impaired mitochondrial distribution and decreased mitochondrial membrane potential in the oocytes of knockout mice. Supplementation with nicotinamide riboside (NR), an NAD^+^ booster, in mutant mice increased ovarian reserve and improved oocyte quality. Our study highlights the importance of the NAD^+^ de novo pathway in middle‐aged female fertility.

## Graphical abstract

The disruption of the de novo nicotinamide adenine dinucleotide (NAD^+^) synthesis pathway through genetic ablation of *Ido1* or *Qprt* led to a reduction in ovarian NAD^+^ levels in aging mice. This reduction resulted in decreased mitochondrial functions, accumulation of reactive oxygen species (ROS), and a decrease in both the quality and quantity of oocytes, accompanied by reduced granulosa cell proliferation, increased apoptosis, and diminished ovarian reserve.

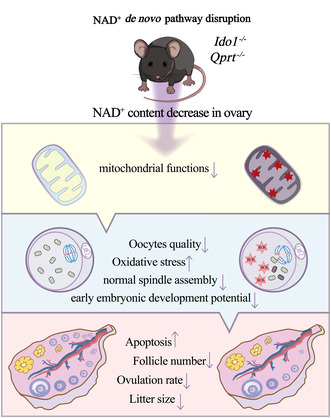

**Abbreviation**
AMH: anti‐Müllerian hormone
COCs: Cumulus–oocyte complexes
DEGs: differentially expressed genes
GCs: Granulosa cells
GSEA: Gene Set Enrichment Analysis
IDO1: indoleamine‐2,3‐dioxygenase 1
IVF: in vitro fertilization
KEGG: Kyoto Encyclopedia of Genes and Genomes
NA: nicotinic acid
NAD+: nicotinamide adenine dinucleotide
NAM: nicotinamide
NFK: N‐formyl kynurenine
NMN: Nicotinamide mononucleotide
NR: nicotinamide riboside
OXPHOS: oxidative phosphorylation
PCA: principal component analysis
PMSG: Pregnant Mares Serum Gonadotropin
QPRT: Quinolinate phosphoribosyl transferase
ROS: reactive oxygen species
TDO: tryptophan‐2,3‐dioxygenase
Trp: amino acid tryptophan

## 1 INTRODUCTION

The ovary is one of the early aging organs in mammals, characterized by a reduction in follicle numbers, a decline in oocyte quality and quantity (Amargant et al., 2020). In humans, female fertility begins to decline around the age of 32 and deteriorates more rapidly after 40, resulting in an increase in oocyte aneuploidies, impaired early embryonic development potential, and a rise in spontaneous abortion rates (Nagaoka et al., 2012). Despite limited knowledge on the underlying molecular mechanisms of ovarian aging, mitochondrial dysfunction has been identified as a hallmark of this process (Chiang et al., 2020). Thus, a deeper understanding of how mitochondria impact ovarian senescence is crucial for the development of therapeutic interventions aimed at improving mitochondrial function and extending the female reproductive lifespan.

Nicotinamide adenine dinucleotide (NAD^+^) is a critical cofactor for a variety of cellular metabolic processes and plays a crucial role in maintaining mitochondrial homeostasis and genome stability (Bonkowski & Sinclair, 2016; Fang et al., 2017).NAD^+^ can be biosynthesized through three different pathways in mammalian cells: de novo from dietary tryptophan through the kynurenine pathway (KP), generation from nicotinic acid (NA) via the Preiss‐Handler (PH) pathway, or synthesis from nicotinamide (NAM) or nicotinamide riboside (NR) through the salvage pathway (SP) (Canto et al., 2015; Xie et al., 2020). The homeostasis of NAD^+^ is carefully regulated through a balance between its biosynthesis and consumption by enzymes, including poly (ADP‐ribose) polymerases (PARPs), sirtuins (SIRT1‐7), cyclic ADP‐ribose synthases, (Canto et al., 2013). Increasing evidence supports that NAD^+^ homeostasis is disrupted in age‐related diseases (Hou et al., 2018; Katsyuba et al., 2020; Sun et al., 2020). We and several other groups have reported that ovarian NAD^+^ levels decline with aging, whereas boosting NAD^+^ by supplementation with NAD^+^ precursors, such as NR or Nicotinamide mononucleotide (NMN), can increase ovarian NAD^+^ levels and mitigate ovarian aging by enhancing mitochondrial function (Bertoldo et al., 2020; Miao et al., 2020; Wang, Yang, et al., 2021; Wu et al., 2019; Yang et al., 2021; Yang, Cong, et al., 2020).

The expression of IDO1 and QPRT, key enzymes in the de novo NAD^+^ biosynthesis pathway, has been previously reported in some other tissues such as the kidney and liver (Houtkooper et al., 2010; Minhas et al., 2019; Xie et al., 2020). However, the role of this pathway in the ovary remains unknown. In this study, we investigated the expression of IDO1 and QPRT in the ovary and assessed the impact of genetic ablation of these two genes on ovarian NAD^+^ levels, ovarian aging, and female fertility. Our results showed that the deletion of IDO1 and QPRT resulted in a decrease in ovarian NAD^+^ levels in middle‐aged mice. This disruption of de novo NAD^+^ synthesis also accelerated ovarian aging, manifested by decreased follicle numbers, impaired oocyte quality, and reduced fertility. Furthermore, supplementation with NAD^+^ booster NR increased ovarian reserve and improved the quality of oocytes, leading to restored fertility. These findings highlight the critical role of the NAD^+^ de novo biosynthesis pathway in female fertility during middle age.

## 2 RESULTS

### 2.1 Disruption of de novo NAD ^+^ synthesis reduced ovarian NAD ^+^ levels and impaired fecundity in aging mice

**FIGURE 1:**
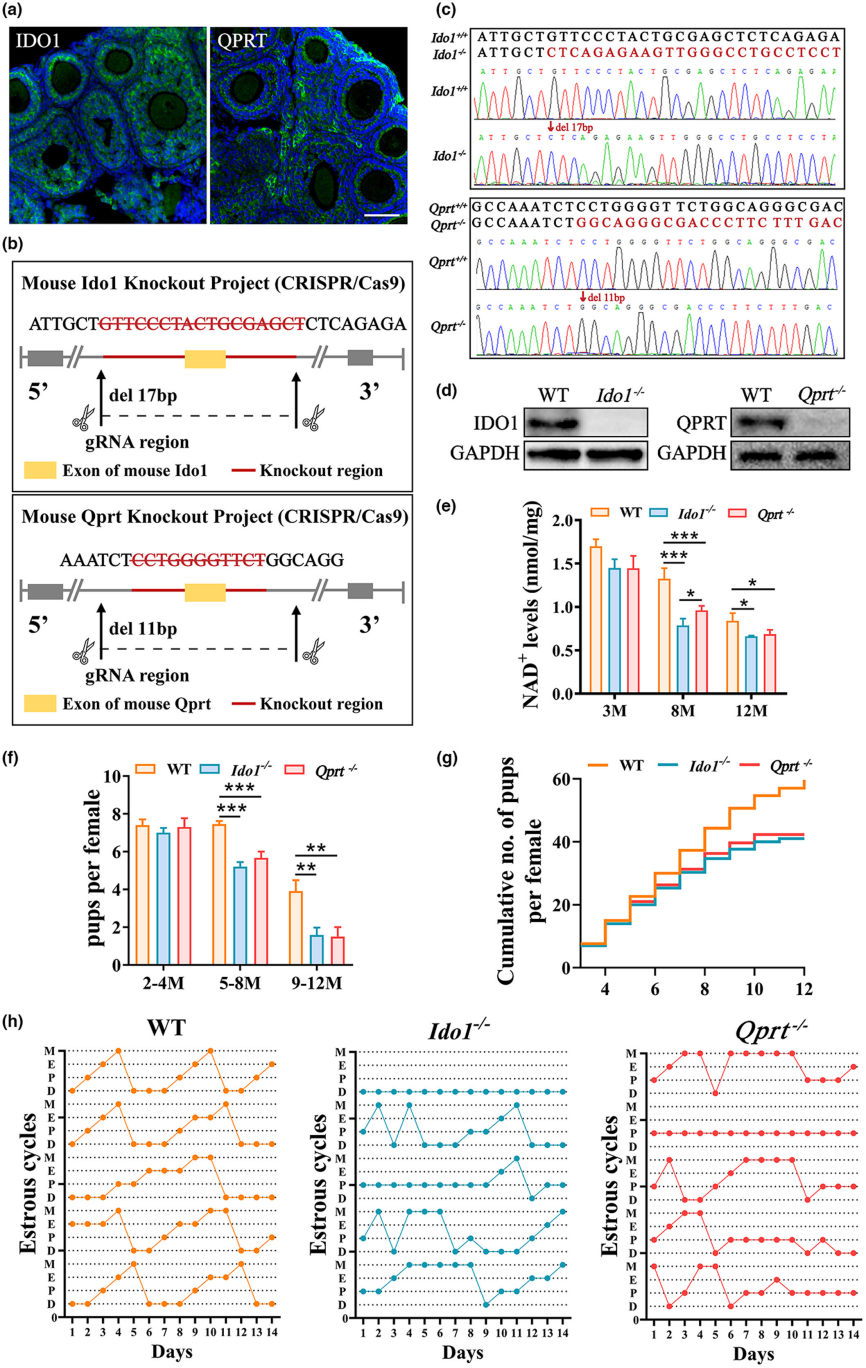
Deletion of *Ido1* or *Qprt* decreased ovarian nicotinamide adenine dinucleotide (NAD^+^) levels and reduced mouse fertility. (a) Representative images of immunofluorescence staining of IDO1 (Left panel, green) and QPRT (right panel, green) and nuclei (blue) in ovarian tissue sections. Scale bar, 50 μm. (b) The schematic diagram of *Ido1* or *Qprt* knockout mouse construction by CRISP/Cas 9. (c) Representative Sanger sequence results of the Ido1 (up panel) and Qprt (down panel) Knockout mice. (d) Western blot confirmed the protein expression in ovaries from WT, *Ido1* and *Qprt* Knockout mice. (e) Detection of ovarian NAD^+^ levels in WT, *Ido1* ^−/−^ and *Qprt* ^−/−^ mice at different ages using the cycling assay (n = 3 mice for each age). (f) The average litter size was evaluated in WT, *Ido1* ^−/−^ and *Qprt* ^−/−^ mice at different ages. (g) Cumulative numbers of pups for each female during the defined time period (n = 3 mice for each genotype). (h) The estruses cyclicity in 8‐month‐old WT, *Ido1* ^−/−^ and *Qprt* ^−/−^ mice (n = 5 mice for each age). *p < 0.05, **p < 0.01, ***p < 0.001.

The gene expressions of rate‐limiting enzymes in de novo NAD^+^ synthesis, *Ido1* and *Qprt* in some organs are shown in Figure S1a,b. Protein expression analysis also showed that both of IDO1 and QPRT are expressed in ovary. To investigate the role of de novo NAD^+^ synthesis pathway on ovarian functions. We generated *Ido1* and *Qprt* knockout mice by using CRISP/Cas9 as shown in Figure 1b. Sanger sequencing was used to confirm the mouse genotypes (Figure 1c). Immunoblotting and immunofluorescence analysis indicated that the IDO1 and QPRT proteins were undetectable in the ovaries from *Ido1* ^−/−^ and *Qprt* ^−/−^ mice, respectively (Figure 1d and Figure S1c,d). The levels of NAD^+^ in the ovaries of WT, *Ido1* ^−/−^ and *Qprt* ^−/−^ mice were measured with aging. Our results indicate that there were no significant differences in NAD^+^ levels among the ovaries of 3‐month‐old WT, *Ido1* ^−/−^ or *Qprt* ^−/−^ mice. However, a notable decline in the NAD^+^ content of ovaries and the isolated granulosa cells was observed in 8 months old *Ido1* ^−/−^ and *Qprt* ^−/−^ mice compared to control mice (Figure 1e and Figure S1e). This alteration was not detected in other tissues investigated (Figure S1f). Furthermore, the fertility test revealed that the average number of pups per female was lower in the *Ido1* ^−/−^ or *Qprt* ^−/−^mice compared with WT controls from the onset of middle age (Figure 1f), resulting in a decrease in the cumulative number of pups in the knockout mice (Figure 1g). These results demonstrate that disruption of de novo NAD^+^ synthesis pathway affects fertility in middle‐aged female mice. Additionally, the estrous cyclicity was found to be irregular in *Ido1* ^−/−^ or *Qprt* ^−/−^ mice compared to age‐matched controls (Figure 1h). These findings suggested that de novo NAD^+^ synthesis pathway is essential for maintaining the ovarian NAD^+^ pool in middle‐aged mice.

### 2.2 Disruption of de novo NAD ^+^ synthesis accelerated follicle loss and ovarian senescence

**FIGURE 2:**
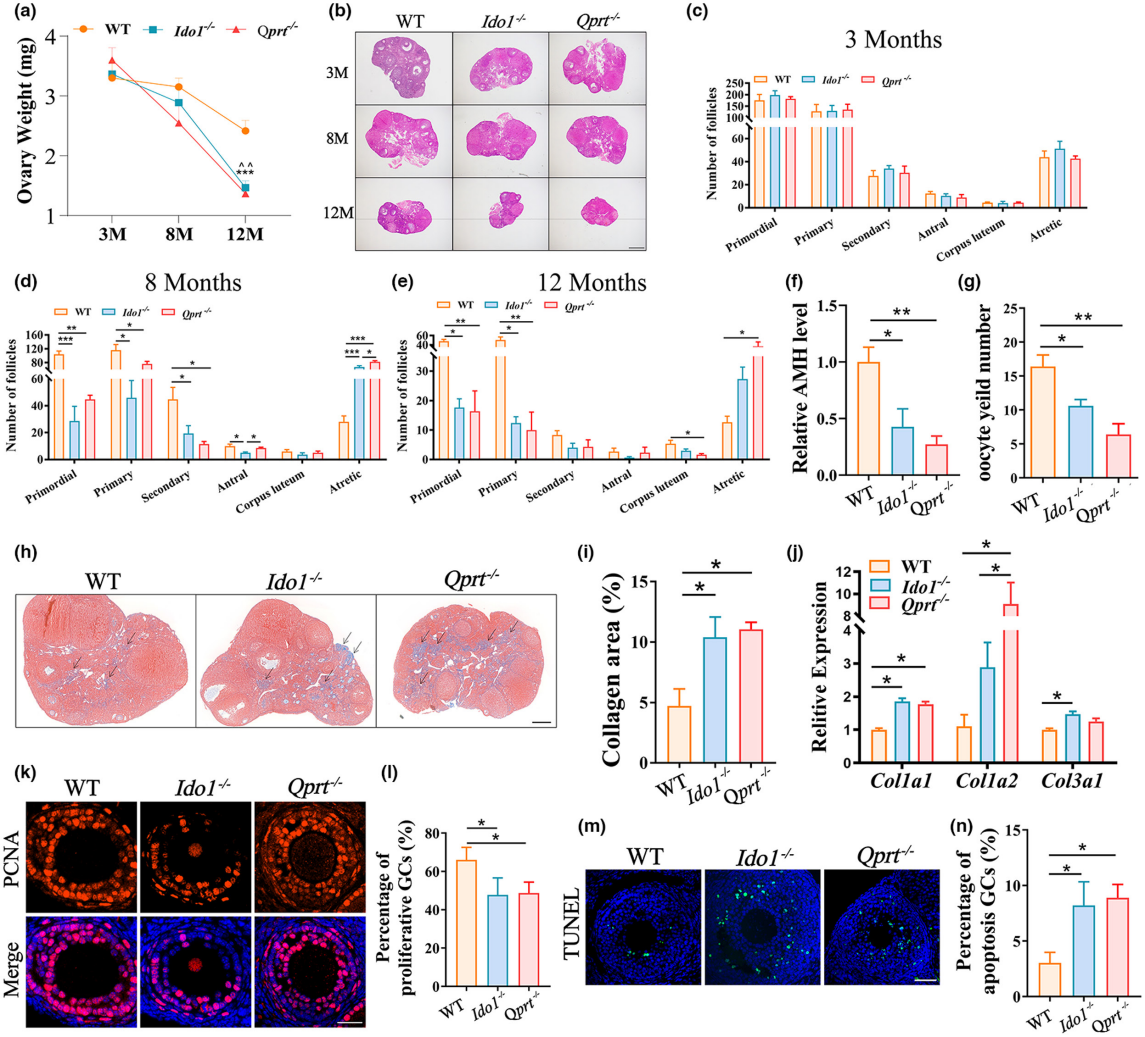
Deletion of *Ido1* or *Qprt* accelerated ovarian senescence. (a) Ovary weight for each group of mice at ages of 3‐, 8‐ and 12‐month (M)‐old (n = 5 mice for each age). ^^: WT VS. *Ido1* ^−/−^, *p* < 0.01; ***: WT VS. *Qprt* ^−/−^, *p* < 0.001. (b) Representative hematoxylin and eosin‐stained ovarian sections from 3,8, and 12‐month (M)‐old WT, *Ido1* ^−/−^ and *Qprt* ^−/−^ mice. Scale bars, 100 μm. (c–e) Quantitative analysis of follicles at different stages in ovarian sections from mice aged 3‐ (c), 8‐ (d) and 12‐month‐old (e) WT, *Ido1* ^−/−^ and *Qprt* ^−/−^ mice (n = 3 mice for each age). (f) Mean serum AMH levels in the 8‐month‐old WT, *Ido1* ^−/−^ and *Qprt* ^−/−^ mice (n = 4 mice for each age). (g) The average number of ovulated oocytes after gonadotropin induction of ovulation in the 8‐month‐old WT, *Ido1* ^−/−^ and *Qprt* ^−/−^ mice (n = 5–8 mice for each group). (h) Ovarian fibrosis examination by using Masson trichrome staining of the ovarian sections from the 8‐month‐old WT, *Ido1* ^−/−^ and *Qprt* ^−/−^ mice. Arrows showed the positive staining. Scale bar, 200 μm. (i) Quantification of the ratio of blue area to total ovarian sections for each group of mice (n = 3 mice for each group). (j) Transcript levels of fibrosis‐related genes (*Col1a1, Col1a2, and Col3a1*) in the ovaries detected by real‐time RT‐PCR from 8‐month‐old WT, *Ido1* ^*−/−*^ and *Qprt* ^*−/−*^ mice (n = 3 mice per group). (k) Representative images of PCNA immunofluorescence staining of ovarian sections from 8‐month‐old WT, *Ido1* ^−/−^ and *Qprt* ^−/−^ mice. Scale bars, 30 μm. (l) Quantification of PCNA‐positive granulosa cells in ovary sections from each group mice (n = 3 mice for each group). (m) Representative images of apoptosis granulosa cells elevated by TUNEL staining from three group mice at the age of 8‐month‐old. Scale bars, 50 μm. (n) Quantitative analysis of TUNEL‐positive granulosa cells in ovarian sections from mice in each group (n = 3 mice for each group). *p < 0.05, **p < 0.01, ***p < 0.001, ^^*p* < 0.0.

To evaluate the roles of IDO1 and QPRT in de novo NAD^+^ synthesis in ovarian aging, the ovarian weights were monitored, and significant decreases were observed in the *Ido1* ^−/−^ or *Qprt* ^−/−^ mice at the age of 12 months old as compared with controls (Figure 2a). The ovarian reserve changes during aging were also assessed by histological staining in ovaries from 3‐, 8‐and 12‐month‐old *Ido1* ^−/−^, *Qprt* ^−/−^ mice and WT littermates. Follicle numbers in ovaries were similar in 3‐month‐old mutant mice and WT mice but significantly decreased in mutant mice at 8 and 12 months of age compared to WT mice (Figure 2b–e). Consistent with decreases in secondary and early antral follicles, serum AMH levels were significantly lower in 8‐month‐old *Ido1* ^−/−^ and *Qprt* ^−/−^ mice compared to WT mice (Figure 2f). In addition, the number of ovulated oocytes was remarkably decreased in the 8‐month‐old mutant mice compared with age‐matched controls (Figure 2g). Fibrosis is a hallmark of ovarian senescence (Briley et al., 2016; Umehara et al., 2022). To evaluate the impact of IDO1 and QPRT on fibrosis in ovarian aging, Masson staining was performed on ovarian sections from mutant mice and wild‐type controls. The staining intensity was markedly higher in the mutant mice than in the wild‐type littermates (Figure 2h,i). Meanwhile, we examined gene expression of cell senescence marker *p21* in ovaries and important organs of 8‐month‐old knockout and WT mice. We found that the expression level of *p21* in ovary of knockout mice was significantly upregulated compared with wild‐type mice, while no significant changes were observed in other tissues tested. (Figure S2). Quantitative analysis was conducted to determine the expression levels of fibrosis‐related genes, including *Col1a1*, *Col1a2*, and *Col3a1*, in 8‐month‐old mice using RT‐PCR. The results showed that the expression levels of *Col1a1*, *Col1a2*, and *Col3a1* were significantly upregulated in the mutant mice compared to the wild‐type controls, providing further evidence of increased fibrosis in the ovaries of mutant mice (Figure 2j). The results indicate that deletion of *de novo* NAD^+^ synthesis accelerated ovarian senescence.

To investigate cellular changes in the ovaries of mutant mice, we examined cell proliferation and apoptosis during follicle development using PCNA and TUNEL staining, respectively. The results showed that granulosa cells in the ovaries of 8‐month‐old *Ido1* ^−/−^ and *Qprt* ^−/−^ mice exhibited lower PCNA‐positive staining compared to age‐matched littermates (Figure 2k,l). Additionally, increased granulosa cell apoptosis was observed in both *Ido1* ^−/−^ and *Qprt* ^−/−^ mice (Figure 2m,n). These results demonstrated that disruption of de novo NAD^+^ synthesis led to a decrease in granulosa cell proliferation and an increase in apoptosis in growing follicles, resulting in follicle loss in aging mice.

### 2.3 Disruption of de novo NAD ^+^ synthesis reduced oocyte quality and impaired the early embryo development potential

**FIGURE 3:**
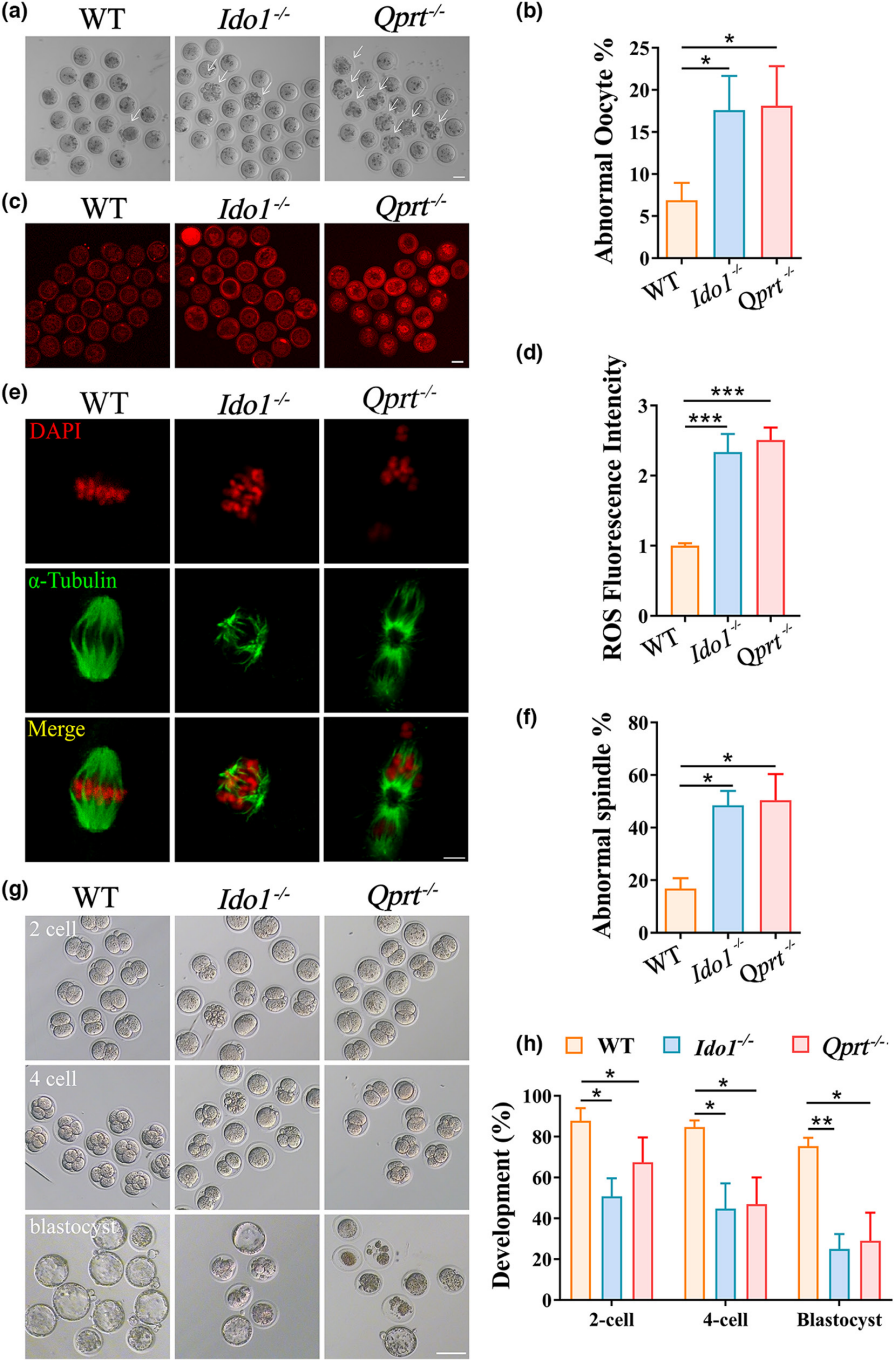
Decreased oocyte quality and lowered embryonic development potential in *Ido1* ^−/−^ and *Qprt* ^−/−^ mice. (a) Representative images of MII oocytes from 8‐month‐old WT, *Ido1* ^−/−^ and *Qprt* ^−/−^ mice. Arrows indicate abnormal oocytes with cytoplasmic fragments. Scale bar, 50 μm. (b) The mean percentage of abnormal oocytes (with cytoplasmic fragments) for each group (n = 5 mice for each group). (c) Representative images of ROS levels detected by Mito‐SOX staining in oocytes from WT, *Ido1* ^−/−^ and *Qprt* ^−/−^ mice at the age of 8‐month‐old. Scale bar, 50 μm. (d) The mean fluorescence pixel intensity of Mito‐SOX in oocytes for each group (n = 32–36 per group). (e) Representative images of spindles and chromosome alignment of MII oocytes from 8‐month‐old WT, *Ido1* ^−/−^ and *Qprt* ^−/−^ mice. Spindles were visualized by staining using an α‐tubulin antibody (green), and chromosomes were displayed by PI staining (red). Scale bar, 10 μm. (f) The rate of aberrant spindles at MII oocytes from each group (n = 36 oocytes for each group). (g) Representative images of 2‐cell embryos, 4‐cell embryos, and blastocyst from 8‐month‐old WT, *Ido1* ^−/−^ and *Qprt* ^−/−^ mice. Scale bar, 50 μm. (h) The rates of 2‐cell embryos, 4‐cell embryos, and blastocyst were recorded for each group. (n = 6 mice for each group). *p < 0.05, **p < 0.01, ***p < 0.0.

To evaluate the impact of disrupting de novo NAD^+^ synthesis on oocyte quality, we examined fragmentation and ROS content in oocytes from 8‐month‐old *Ido1* ^−/−^ and *Qprt* ^−/−^ mice. As shown in Figure 3a,b, oocytes from mutant mice exhibited increased fragmentation compared to age‐matched WT controls. Mito‐SOX staining revealed that ROS content was approximately 2.5‐fold higher in MII oocytes from 8‐month‐old mutant mice compared to WT controls (Figure 3c,d). Additionally, we used anti‐tubulin antibody staining to visualize spindles and propidium iodide staining to localize DNA in chromosomes. As shown in Figure 3e,f, approximately 50% and 60% of MII oocytes from 8‐month‐old *Ido1* ^−/−^ and *Qprt* ^−/−^ mice exhibited abnormal spindle morphology, along with chromosome misalignment. In contrast, about 20% of oocytes showed these meiotic defects in WT controls. Furthermore, we conducted in vitro fertilization (IVF) of MII oocytes from different groups of mice at 8 months old to test the developmental capacity of early embryos derived from the mutant mice. The results showed that the fertilization rate was lower in oocytes from mutant mice compared to WT controls, and the rates for 4‐cell and blastocyst embryo formation decreased dramatically in mutant mice compared to WT controls (Figure 3g,h). These findings suggested that de novo NAD^+^ synthesis is crucial for oocyte development by maintaining redox balance, normal meiosis, oocyte fertilization, and embryonic development.

### 2.4 Disruption of NAD ^+^ de novo pathway resulted in ovarian mitochondrial dysfunction

**FIGURE 4:**
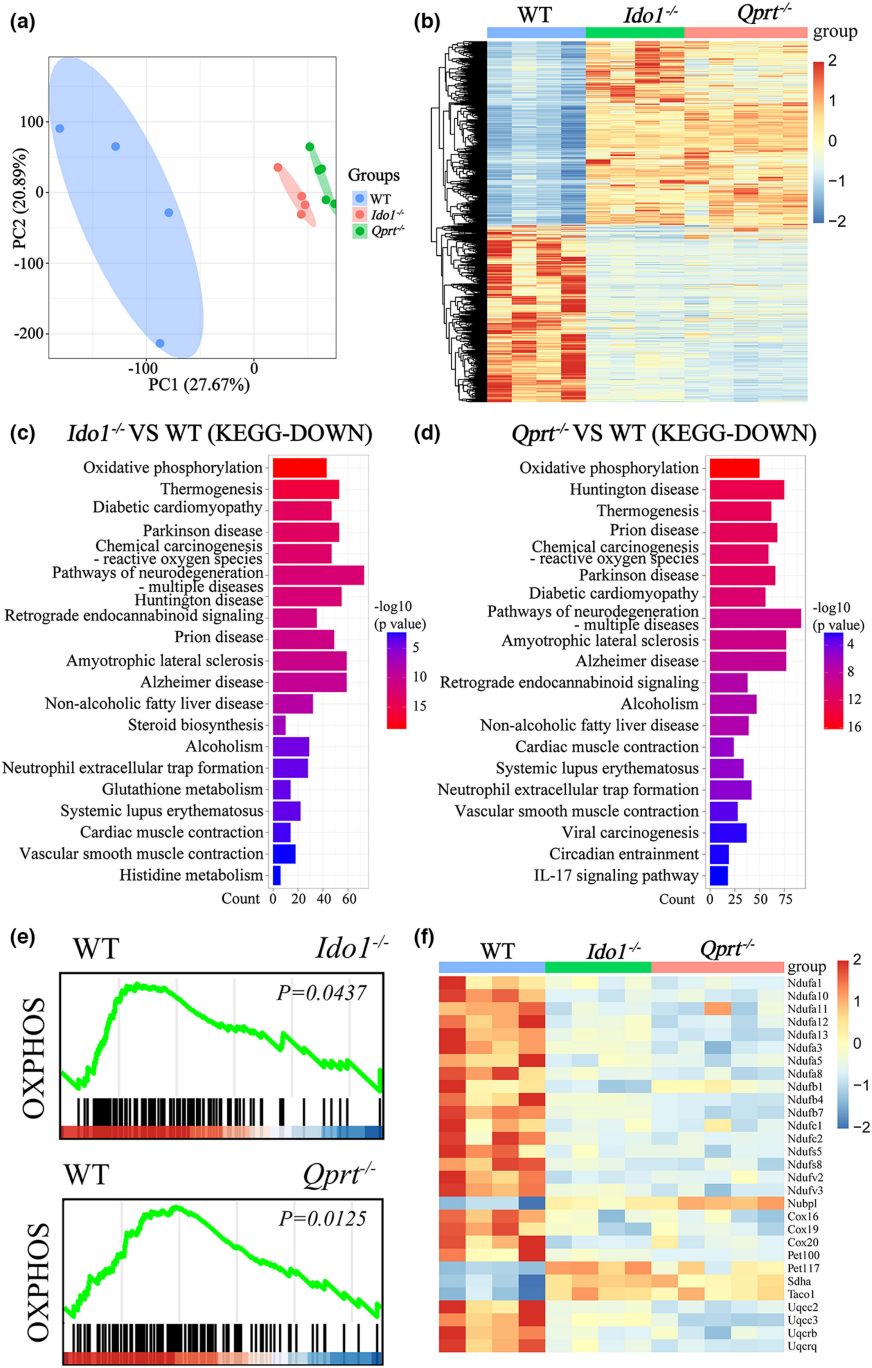
OXPHOS pathway was significantly suppressed in *Ido1* ^−/−^ and *Qprt* ^−/−^ mice ovaries. (a) Principal component analysis displays the gene expression profile cluster in 8‐month‐old WT, *Ido1* ^−/−^ and *Qprt* ^−/−^ mice. (b) Heatmap showing genes differentially expressed in the ovaries of 8‐month‐old *Ido1* ^−/−^ and *Qprt* ^−/−^ mice compared with WT controls. (c, d) Pathway enrichment using KEGG analysis of downregulated DEGs in ovaries from *Ido1* ^−/−^ (c) and *Qprt* ^−/−^ mice (d) compared with WT mice. The color represents *p* value, and the x‐axis showed the gene number of downregulated genes from each KEGG annotation among the DEGs. (e) GSEA analysis showing enrichment of suppressed OXPHOS in ovaries from *Ido1* ^−/−^ and *Qprt* ^−/−^ mice compared with WT mice. (f) Heatmap analysis showing the DEGs in the OXPHOS pathway in *Ido1* ^−/−^ and *Qprt* ^−/−^ mice ovaries.

To investigate the mechanisms underlying the reduced fertility caused by disruption of the NAD^+^ de novo pathway, we conducted transcriptome analysis of ovaries from 8‐month‐old *Ido1* ^−/−^ and *Qprt* ^−/−^ mice, as well as WT mice. Principal Component Analysis (PCA) and Heatmap analyses revealed that the transcriptome profile of ovaries after deletion of *Ido1* or *Qprt* was different from that of age‐matched controls (Figure 4a,b). Compared to WT mice, a total of 1792 genes were significantly upregulated in *Ido1* ^*−/−*^ and *Qprt* ^*−/−*^ mice, whereas 1766 genes were downregulated. Some of the differentially expressed genes (DEGs) in the *Ido1* ^−/−^ and *Qprt* ^−/−^ mice, identified through RNA sequencing, were verified by RT‐PCR (Figure S3). Kyoto Encyclopedia of Genes and Genomes (KEGG) analysis of the DEGs revealed that downregulated genes enriched in the oxidative phosphorylation (OXPHOS) pathway were ranked No. 1 in both ovaries of *Ido1* ^−/−^ and *Qprt* ^−/−^ mice, compared to controls (Figure 4c,d), suggesting that OXPHOS was suppressed in the ovary after disruption of NAD^+^ de novo pathway. Furthermore, Gene Set Enrichment Analysis (GSEA) showed enrichment of suppressed OXPHOS pathway in *Ido1* ^−/−^ and *Qprt* ^−/−^ ovaries (Figure 4e). Heatmap analysis showed that most of the genes in this OXPHOS pathway were down‐regulated in *Ido1* ^−/−^ and *Qprt* ^−/−^ ovaries compared to controls (Figure 4f).

**FIGURE 5:**
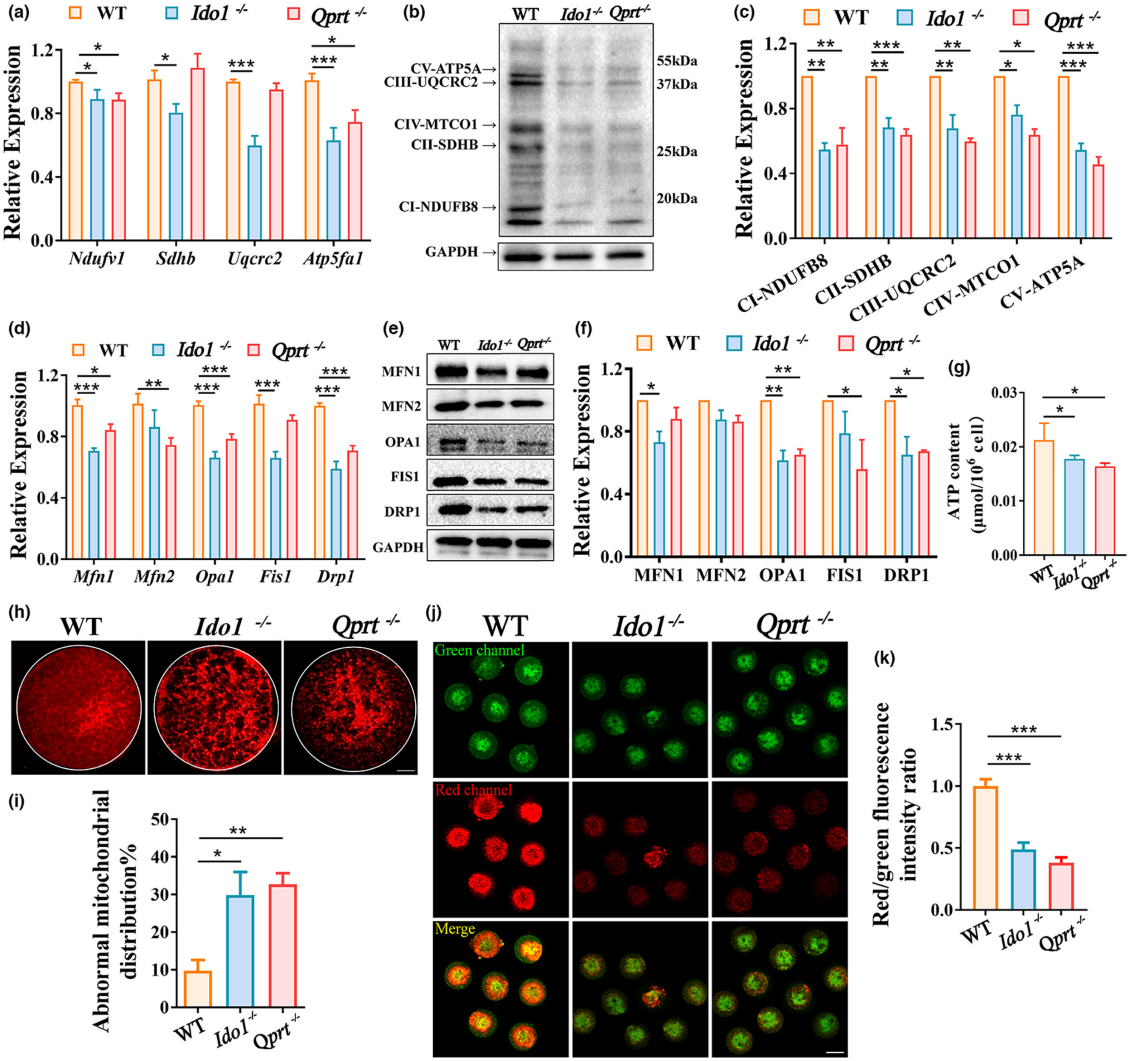
Deletion of *Ido1* or *Qprt* led to ovarian mitochondrial dysfunctions. (a) Transcript levels of mitochondrial electron transport chain complex genes (CI, *Ndufv1*; CII, *Sdhb*; CIII, *Uqcrc2*; CV, *Atp5a1*) in the ovaries detected by real‐time RT‐PCR from 8‐month‐old WT, *Ido1* ^−/−^ and *Qprt* ^−/−^ mice (n = 4 mice per group). (b) Western blot analysis of mitochondrial electron transport chain complex protein expression (CI, NDUFB8; CII, SDHB; CIII, UQCRC2; CIV, MTCO1; CV, ATP5A) in ovaries from 8‐month‐old WT, *Ido1* ^−/−^ and *Qprt* ^−/−^ mice. (c) Relative expression of each protein was assessed as a ratio to GAPDH levels in each lane (n = 3 mice for each group). (d) Transcription analysis of essential dynamic genes for mitochondrial fusion (*Mfn1, and Mfn2, Opa1*) and fission (*Drp1 and Fis1*) (n = 4 mice for each group) in the ovaries from 8‐month‐old WT, *Ido1* ^−/−^ and *Qprt* ^−/−^ mice. (e) Immunoblotting for mitochondrial fusion and fission proteins for each group. (f) Relative expression of each protein was assessed as a ratio to GAPDH levels in each lane (n = 3 mice for each group). (g) ATP content of granulosa cells from 8‐month‐old WT, *Ido1* ^−/−^ and *Qprt* ^−/−^ mice. (h) Mitochondrial distribution was assessed by staining Mito‐tracker in the oocytes from 8‐month‐old WT, *Ido1* ^−/−^ and *Qprt* ^−/−^ mice. Scale bars, 10 μm. (i) The abnormal mitochondrial distribution pattern percentages in oocytes from 8‐month‐old WT, *Ido1* ^−/−^ and *Qprt* ^−/−^ mice (n = 42 oocytes for WT mice, n = 34 oocytes for *Ido1* ^−/−^ mice, n = 37 oocytes for *Qprt* ^−/−^ mice). (j) Mitochondrial membrane potential was determined using JC‐1 staining in oocytes from 8‐month‐old WT, *Ido1* ^−/−^ and *Qprt* ^−/−^ mice. Red fluorescence indicated high mitochondrial membrane potential, and green signals indicated low mitochondrial membrane potential. Scale bar, 50 μm. (k) Average ratios of red to green fluorescence in oocytes from each group (n = 7 oocytes for WT mice, n = 8 oocytes for *Ido1* ^−/−^ mice, n = 10 oocytes for *Qprt* ^−/−^ mice). *p < 0.05, **p < 0.01, ***p < 0.001.

Considering OXPHOS pathway is highly dependent on the proteins for the electron transport chain (Aguilar‐Lopez et al., 2020; Frazier & Thorburn, 2012), we examined the expression of CI (Ndufv1), CII (Sdhb), CIII (Uqcrc2), and CV (Atp5a1) in the ovaries from 8 months old mice. Results showed that the expression of these genes and proteins was decreased in ovaries from *Ido1* ^−/−^ and *Qprt* ^−/−^ mice compared to controls (Figure 5a–c). As mitochondria dynamics are closely related to OXPHOS activities (Yao et al., 2019; Zhou et al., 2022), we conducted RT‐PCR to determine the transcription of genes involved in mitochondrial fission (*Drp1* and *Fis1*) and fusion (*Mfn1*, *Mfn2*, and *Opa1*). Our results showed decreased expression of these mitochondrial fusion and fission genes in the ovaries of *Ido1* ^−/−^ and *Qprt* ^−/−^ mice compared to age‐matched controls (Figure 5d). Furthermore, Western blot analysis revealed a decrease in the expression of mitochondrial fission and fusion proteins in the ovaries of knockout mice (Figure 5e,f). Consequently, the ATP levels in isolated granulosa cells of mutant mice were lower than those of age‐matched controls (Figure 5g).

Furthermore, mitochondrial distribution was analyzed in MII oocytes by staining with Mitotracker. We observed a higher proportion of mitochondria with aggregated cluster distribution in the *Ido1* ^−/−^ and *Qprt* ^−/−^ oocytes, while mitochondria were evenly distributed in the cytoplasm of WT oocytes (Figure 5h,i). Mitochondrial membrane potential (ΔΨm) was also evaluated in the oocytes using the inner membrane potential dye, 5, 5′, 6, 6′‐tetrachloro‐1, 1′, 3, 3′‐tetraethylbenzimidazolylcarbocyanine iodide (JC‐1). We found that mitochondrial membrane potential was decreased in *Ido1* ^−/−^ and *Qprt* ^−/−^ oocytes compared to normal controls (Figure 5j,k). Collectively, our data indicated that disruption of the NAD^+^ de novo pathway results in ovarian mitochondrial dysfunction, primarily by repressing OXPHOS.

### 2.5 Supplementation with NR elevated ovarian NAD ^+^ levels and recused female fertility in aging *Ido1* ^−/−^ and *Qprt* ^−/−^ mice

**FIGURE 6:**
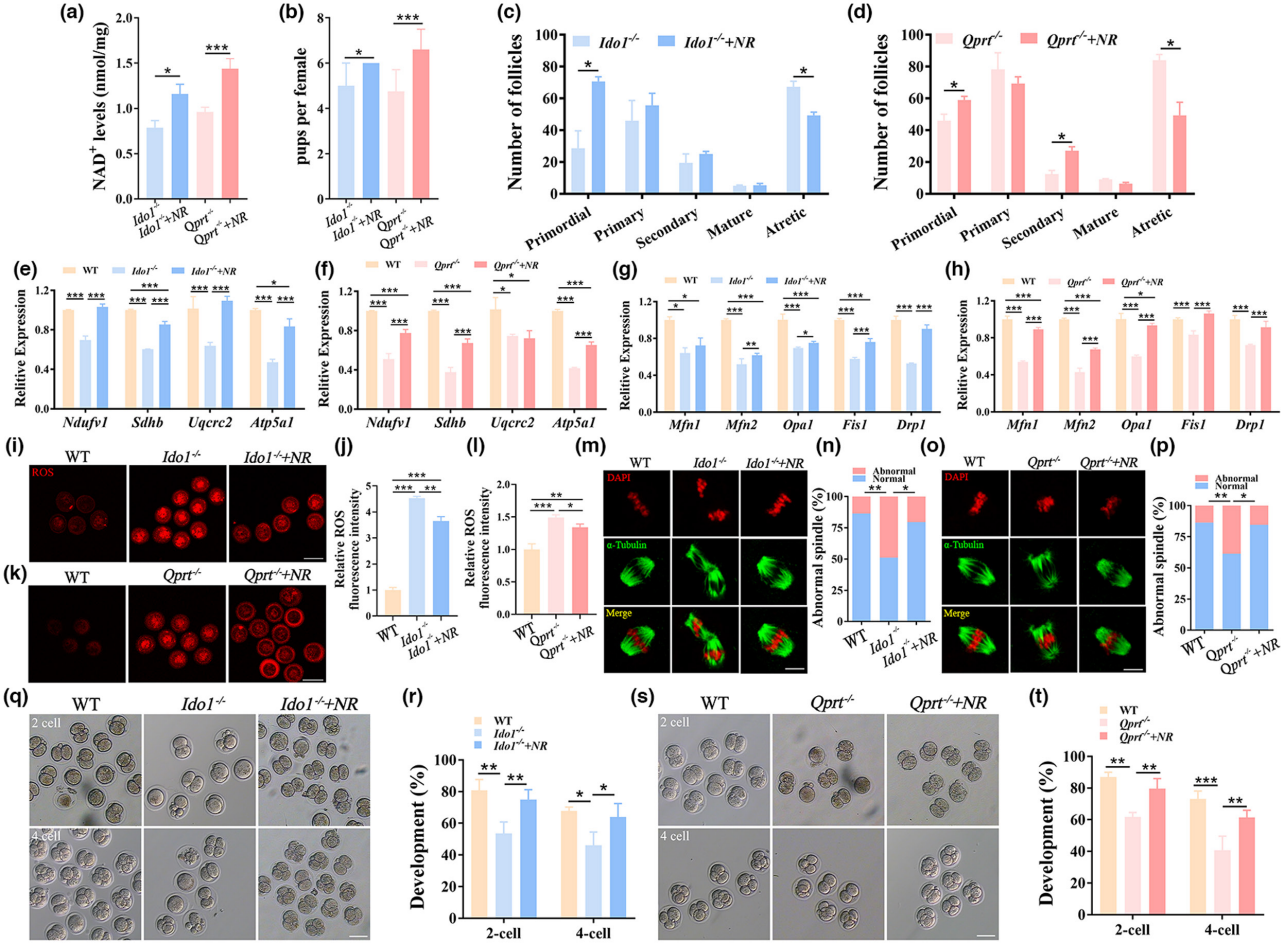
NR supplementation increased ovarian NAD^+^ levels and recused female fertility in aging *Ido1* ^−/−^ and *Qprt* ^−/−^ mice. (a) NAD^+^ levels were determined in ovaries from 8‐month‐old *Ido1* ^−/−^, *Qprt* ^−/−^ mice with or without supplementation of NR at the age of 8‐month‐old (n = 3 mice for each group). (b) The mean pups were recorded in *Ido1* ^−/−^, *Qprt* ^−/−^ mice after supplementation of NR compared with controls (n = 3 mice for each group). (c, d) The follicle numbers were determined for each stage in the ovaries at the age of 8‐month‐old *Ido1* ^−/−^, *Ido1* ^−/−^ + NR mice (c) and *Qprt* ^−/−^, *Qprt* ^−/−^ + NR mice (d) (n = 3 mice for each group). (e, f) Transcript levels of mitochondrial electron transport chain complex genes (CI, *Ndufv1*; CII, *Sdhb*; CIII, *Uqcrc2*; CV, *Atp5a1*) in the ovaries detected by real‐time RT‐PCR from 8‐month‐old *Ido1* ^−/−^, *Ido1* ^−/−^ + NR mice (e) and *Qprt* ^−/−^, *Qprt* ^−/−^ + NR mice (f) (n = 3 mice per group). (g, h) Transcription analysis of essential dynamic genes for mitochondrial fusion (*Mfn1, and Mfn2, Opa1*) and fission (*Drp1 and Fis1*) in the ovaries from 8‐month‐old *Ido1* ^−/−^, *Ido1* ^−/−^ + NR mice (g) and *Qprt* ^−/−^, *Qprt* ^−/−^ + NR mice (h) (n = 3 mice for each group). (i) Representative images of ROS levels detected by Mito‐SOX staining in oocytes from 8‐month‐old WT, *Ido1* ^−/−^ and *Ido1* ^−/−^ mice after supplementation of NR. (j) The mean fluorescence pixel intensity of Mito‐SOX in oocytes for WT, *Ido1* ^−/−^, *Ido1* ^−/−^ + NR mice (n = 6–10 oocytes for each group). (k) Representative images of ROS levels detected by Mito‐SOX staining in oocytes from 8‐month‐old WT, *Qprt* ^−/−^ mice with or without NR supplementation at the age of 8‐month‐old. Scale bars, 100 μm. (l) The mean fluorescence pixel intensity of Mito‐SOX in oocytes for WT, *Qprt* ^−/−^ mice and *Qprt* ^−/−^ + NR mice (n = 8–10 oocytes). (m) Representative images of spindles and chromosome alignment of MII oocytes from 8‐month‐old WT, *Ido1* ^−/−^ and *Ido1* ^−/−^ mice after supplementation of NR. Scale bar, 10 μm. (n) The rate of aberrant spindles at MII oocytes from each group (n = 25–27 oocytes for each group). (o) Representative images of spindles and chromosome alignments of MII oocytes from 8‐month‐old WT, *Qprt* ^−/−^, and *Qprt* ^−/−^ + NR mice. (p) The rate of aberrant spindles at MII oocytes from each group (n = 25–28 oocytes for each group). (q) Representative images of 2‐cell and 4‐cell embryos from 8‐month‐old WT and *Ido1* ^*−/−*^ mice with or without supplementation of NR (0.5 μM) in culture medium. Scale bar, 50 μm. (r) The rates of 2‐cell and 4‐cell embryos were recorded for each group (n = 4 mice for each group). (s) Representative images of 2‐cell and 4‐cell embryos from 8‐month‐old WT and *Qprt* ^*−/−*^ mice with or without supplementation of NR (0.5 μM) in culture medium. Scale bar, 50 μm. (t) The rates of 2‐cell and 4‐cell embryos were recorded for each group (n = 4 mice for each group). *p < 0.05, **p < 0.01, ***p < 0.001.

We conducted further tests to examine the effect of increasing ovarian NAD^+^ levels through supplementation with NR in *Ido1* ^−/−^ and *Qprt* ^−/−^ mice. And 4‐week‐old mice were given NR supplementation at 400 mg/kg body weight/day by feeding with food for 7 months, as previously described (Camacho‐Pereira et al., 2016; Cantó et al., 2012; Cercillieux et al., 2022; Lauritzen et al., 2021; Vignier et al., 2018; Yaku et al., 2021; Yang et al., 2021). As expected, a significant increase in NAD^+^ level was observed in the ovaries of knockout mice after 7 months of NR supplementation compared to age‐matched controls (Figure 6a). Importantly, the mean litter size for both knockout mice was markedly increased after NR supplementation (Figure 6b), indicating that NR supplementation partially restored fertility in *Ido1* ^−/−^ and *Qprt* ^−/−^ mice. Ovarian reserve was also investigated by counting the follicle number at different stages in the ovarian sections. We observed an increase in primordial follicle and a decrease in atretic follicle number in the ovaries from 8‐month‐old *Ido1* ^−/−^ and *Qprt* ^−/−^ mice after NR treatment compared to controls (Figure 6c,d). Most of the genes detected related to mitochondrial dynamics and the mitochondrial electron transport chain complex were evaluated in the ovaries of mutant mice following NR supplementation. These results indicated that most of these genes were upregulated after NR supplementation (Figure 6e–h).

To determine if oocyte quality could be rescued, mitochondrial ROS levels were monitored in MII oocytes from the knockout mice after NR supplementation. Mitochondrial ROS levels were significantly decreased in the oocytes of knockout mice with NR supplementation compared to controls (Figure 6i–l). We then assessed spindle assembly using confocal analysis of α‐tubulin and DNA distribution in the MII oocytes from NR‐treated knockout mice. Meiotic abnormalities, including abnormal spindle and chromosome alignments, were largely decreased in the knockout mice after supplementation with NR (Figure 6m–p). Furthermore, we also investigate the effect of embryonic development of oocytes from *Ido1* ^−/−^ and *Qprt* ^−/−^ mice treated by supplemented with NR in vitro, we found that supplementation of NR also increased the embryonic development potential (Figure 6q–t). These results demonstrated that NR supplementation ameliorate mutant ovarian aging by increasing ovarian NAD^+^ levels.

## 3 DISCUSSION

Our previous studies, as well as others, have shown that the level of NAD^+^ in the ovary is closely related to fertility in aging female mice (Bertoldo et al., 2020; Miao et al., 2020; Yang, Cong, et al., 2020). However, the cellular mechanisms underlying the maintenance of NAD^+^ levels in the ovary with aging are still unknown. Here, we report that the NAD^+^ de novo pathway plays a vital role in maintaining ovarian NAD^+^ levels during the onset of middle age when ovarian NAD^+^ levels decline in mice. This is reflected in lower ovarian NAD^+^ levels in mice at 8 months of age after disruption of the NAD^+^ de novo pathway by deletion of *Ido1* or *Qprt*, which resulted in a decrease in ovarian reserve and oocyte quality, as well as early embryonic development potential. However, all of these changes could be largely reversed by supplementation with NR. Mechanistically, our study showed that deletion of *Ido1* or *Qprt* accelerates ovarian aging by perturbing oxidative phosphorylation (OXPHOS).

NAD^+^ can be synthesized through three pathways, namely the NAD^+^ Preiss‐Handler pathway, NAD^+^ salvage pathway, and NAD^+^ de novo pathway (Amjad et al., 2021; Liu et al., 2018). These biosynthetic pathways play a critical role in maintaining NAD^+^ pools, which are vital to cellular energy and metabolism (Amjad et al., 2021). Previous studies have suggested that the NAD^+^ de novo biosynthesis pathway primarily occurs in the liver and kidneys, whereby the amino acid tryptophan (Trp) is catabolized through the kynurenine (KYN) pathway (Bignon et al., 2022; Fletcher & Lavery, 2018). The first rate‐limiting step involves the oxidation of Trp to N‐formyl kynurenine (NFK) by either indoleamine‐2,3‐dioxygenase (IDO) or tryptophan‐2,3‐dioxygenase (TDO), followed by the second rate‐limiting step of catalyzing the formation of NAMN from QA by Qprt (Badawy, 2017). In the present study, we observed that the rate‐limiting enzymes IDO1 and QPRT, involved in the NAD^+^ de novo pathway, were mainly localized in the cytoplasm of oocytes and granulosa cells. We found that the ovarian NAD^+^ content decreased in middle‐aged mice after deletion of *Ido1* and *Qprt*, leading to reduced oocyte fertilization ability, early embryo developmental potential, and accelerated ovarian aging, as evidenced by irregular estrous cycles and decreased offspring sizes. Our data demonstrated that the NAD^+^ de novo pathway plays an essential role in determining fertility in aged female mice by maintaining NAD^+^ levels.

Furthermore, we observed that disruption of the NAD^+^ de novo pathway impaired spindle assembly in oocytes. Previous studies have demonstrated that knockdown or inhibition of NAMPT, a rate‐limiting enzyme for NAD^+^ synthesis in the salvage pathway, resulted in reduced NAD^+^ levels in oocytes and led to severely compromised division asymmetry during oocyte maturation by disturbing spindle assembly (Diaz Brinton, 2012; Wang, Zhu, et al., 2021; Wei et al., 2020). These findings suggest that sustained NAD^+^ levels are necessary for proper spindle assembly during oocyte meiosis. Notably, advanced maternal age is associated with a significant increase in meiotic chromosome segregation errors, leading to aneuploidy in oocytes, which is considered a major factor responsible for the increased incidence of age‐related infertility (Battaglia et al., 1996; Mikwar et al., 2020; Selesniemi et al., 2011). Studies have indicated that defects in the organization of the meiotic spindle are a leading cause of age‐related aneuploidy in oocytes (Charalambous et al., 2022; Duncan et al., 2009; Holubcova et al., 2015). Collectively, these results suggest that NAD^+^ deficiency‐induced abnormal spindle assembly may be a crucial factor for age‐associated aneuploidy in oocytes. Therefore, increasing NAD^+^ levels through supplementation with NAD^+^ precursors may represent a potential therapeutic approach for treating age‐related aneuploidy in oocytes.

In order to elucidate the molecular mechanisms by which NAD^+^ de novo pathway regulates fertility in aging females, we performed transcriptome profiling to identify potential targets. Our findings revealed that the expression of genes related to mitochondrial OXPHOS was significantly downregulated in the ovaries of 8‐month‐old knockout mice as compared to controls, suggesting that disruption of NAD^+^ de novo pathway resulted in mitochondrial dysfunction which was mediated by the suppression of OXPHOS. Our results support the notion that impaired mitochondrial functions lead to ovarian senescence (Cecchino et al., 2021; May‐Panloup et al., 2016; van der Reest et al., 2021; Yang, Lin, et al., 2020). Subsequent analysis revealed that deletion of *Ido1* or *Qprt* impaired mitochondria distribution and decreased mitochondrial membrane potential in oocytes. Mitochondrial dysfunction is known to ROS production (Kudryavtseva et al., 2016; Murphy, 2013). Accordingly, ROS levels were significantly elevated in *Ido1* ^−/−^ and *Qprt* ^−/−^ oocytes. Mitochondrial OXPHOS is known to play a crucial role in maintaining the function of the mitotic spindle in oocytes (Zhang et al., 2006). Impairment of OXPHOS leads to a reduction in energy production and an accumulation of ROS, which can result in spindle abnormalities, including improper microtubule attachment, incorrect spindle orientation, and improper chromosome segregation (Al‐Zubaidi et al., 2021; Combelles et al., 2009; Wang et al., 2019; Zhao et al., 2022). Our data demonstrate that the disruption of the NAD^+^ de novo pathway leads to disturbed mitochondrial functions, suppressed granulosa cell proliferation, and decreased oocyte quality, ultimately resulting in reduced fertility. These findings provided further support for the idea that disrupted mitochondrial functions are a key contributor to ovarian senescence (Cecchino et al., 2021; May‐Panloup et al., 2016; van der Reest et al., 2021; Yang, Lin, et al., 2020).

In conclusion, this study provides in vivo evidence that disruption of NAD^+^ de novo pathway results in reduced ovarian NAD^+^ levels, leading to mitochondrial dysfunction and decreased ovarian reserve and oocyte quality in aging mice. These findings enhanced our understanding of the mechanisms underlying middle‐aged female fertility and the crucial role of adequate supplementation of NAD^+^ de novo pathway precursor in counteracting age‐related ovarian infertility.

## 4 MATERIALS AND METHODS

### 4.1 Animals' feeding and treatment

The Ido1 and Qprt knockout mice were generated using CRISPR/Cas9‐mediated genome editing technology from Cyagen. Genomic DNA was extracted from the mouse tails using a DNA extraction kit (Qiagen) and subjected to Sanger DNA sequencing to confirm the homozygous knockout status. For the supplementation treatment, four‐week‐old *Ido1* ^*−/−*^ and *Qprt* ^*−/−*^ mice were fed with nicotinamide riboside (NR, Shanghai Biochempartner Co., Ltd) at a dose of 400 mg/kg/day for 7 months. Wild‐type C57/BL6 mice were purchased from Beijing Vital River Experimental Animals Centre. The mice were housed under a 12‐h light–dark cycle and maintained at a room temperature of 20–25°C with access to food and water ad libitum.

### 4.2 Measurement of NAD ^+^ level in the ovary

The measurement of NAD^+^ levels was performed using the NAD/NADH Assay Kit (Abcam) as per the manufacturer's instructions. The ovarian tissue was homogenized in lysis buffer and the supernatant was collected after centrifugation. The samples and the NAD^+^ Extraction buffer were pre‐heated at 37°C for 10 min. Then, the NADH Extraction and NADH Reaction Mixture were added to the tissue supernatant and incubated at room temperature for 2 h. Subsequently, the fluorescence intensity was determined in a microplate reader with excitation and emission wavelengths set at 540/590 nm.

### 4.3 Oocyte collection and in vitro fertilization

Female mice were subjected to intraperitoneal injection of 7.5 IU Pregnant Mares Serum Gonadotropin (PMSG, Solarbio) followed by 7.5 IU Human Chorionic Gonadotropin (hCG, Solarbio) 48 h later to induce superovulation. After 14‐16 h, the mice were euthanized, and the oviducts were removed. Cumulus‐oocyte complexes (COCs) were retrieved from the oviductal ampullae in M2 medium (Nanjing Aibei Biotechnology). Granulosa cells (GCs) were obtained by puncturing the ovaries in M2 medium containing 1% hyaluronidase.

For in vitro fertilization, COCs were transferred to the fertilization medium (COOK). Male mice 3‐month‐old were sacrificed, and epididymites were removed. The spermatozoa were released from dissected epididymis in an HTF medium (Nanjing Aibei Biotechnology). After capacitation for 1 h at 37°C in a 5% CO2 incubator, the spermatozoa were added to the fertilization medium and allowed to remain for 6 h. Oocytes with two pronuclei, indicating successful fertilization, were moved to the KSOM medium (Nanjing, Aibei Biotechnology). The rates of embryo formation rates in different stages were monitored.

### 4.4 Determination of ROS levels in oocytes

Oocytes from different groups were incubated in M2 medium with 5 μm MitoSOX (Thermo Fisher Scientific) at 37°C in an atmosphere of 5% CO_2_ incubator for 20 min in the dark. After washing three times with M2 medium, the oocytes were imaged using a laser scanning confocal microscope (Zeiss LSM 700). The Image J software was used to calculate the average fluorescence intensity in oocytes for all experimental groups.

### 4.5 Measurement of mitochondrial membrane potential and distribution in oocytes

For the mitochondrial membrane potential assay, the oocytes from various groups were subjected to incubation with 10 μM JC‐1 (Beyotime Biotechnology) in a 100 μL working solution supplied by the kit at 37°C in 5% CO_2_ for 20 min. Following three washes in PBS, the confocal microscope (Zeiss LSM 700) was utilized to measure the red and green fluorescence intensities in each oocyte. The red/green fluorescence intensity signifies the mitochondrial membrane potential.

To conduct the oocyte mitochondrial distribution assay, the MII oocytes from each group were incubated in M2 medium with 250 nm MitoTracker Red (Thermo Fisher Scientific) at 37°C in 5% CO_2_ for 30 min. After washing three times in the M2 medium, the oocytes were observed using the laser scanning confocal microscope (Zeiss LSM 700). The Image J software was used to calculate the fluorescence intensity.

### 4.6 Immunofluorescence

The oocytes were fixed with a 4% paraformaldehyde solution for 30 min and subsequently permeabilized using 0.5% Triton X‐100 in PBS for 20 min. After multiple washes with PBS, the oocytes were blocked using 1% BSA for 1 h. The oocytes were then incubated overnight at 4°C with the anti‐α‐tubulin monoclonal antibody (1:120, Sigma) to visualize the spindle. After washing several times with PBS, the oocytes were incubated with Alexa Fluor 488 goat anti‐mouse secondary antibody (Invitrogen, California, USA) in the dark for 1 h at 37°C. Subsequently, the oocytes were counterstained with 10 μg/mL propidium iodide for 10 min and then placed onto glass slides for imaging using a confocal microscope (Zeiss LSM 700).

### 4.7 Measurement of serum anti‐Müllerian hormone (AMH) levels

Blood was taken from the inner canthus vein of mice, and serum was collected. Mouse serum AMH level was measured for each group using a chemiluminescence immunoassay kit (Roche Diagnostics, Basel, Switzerland) according to the manufacturer's introduction on a Roche Diagnostics Cobas 6000 analyzer.

### 4.8 Monitoring of estrous cycles

Vaginal smears were taken from 8‐month‐old mice for 14 consecutive days. The smears were stained with hematoxylin and eosin and evaluated under the microscope (Nikon). Estrous cycle stages were determined based on criteria described previously (Byers et al., 2012).

### 4.9 ATP content assessments

Granulosa cells were isolated from freshly removed ovaries of C57BL/6 mice from each group, and the extraction procedure was performed in accordance with the manufacturer's instructions. Briefly, the corresponding volume of extraction buffer was added to the cell suspension and mixed thoroughly and centrifugation at 10000*g* for 10 min. Take the supernatant and mix it with same volume of chloroform thoroughly before centrifugation, and the supernatant was collected for the test. The absorbance at 340 nm was measured and the ATP content was calculated according to the instructions.

### 4.10 Quantitative real‐time PCR

Ovarian total RNA was extracted using the Trizol method. The RNA was then reverse transcribed into cDNA using the HIScript III RT SuperMix for qPCR (Vazyme), following the manufacturer's instructions. Quantitative real‐time PCR was performed on a Quantstudio 12 K Flex (Applied Biosystems) using the SYBR qPCR Master Mix (Vazyme). The relative gene expression levels were determined by normalizing to *gapdh* levels. The primer sequences used are listed in Table S1.

### 4.11 Western blot

Ovarian proteins were extracted using protein lysis buffer (Sangon Biotech, Shanghai, China) and quantified using a protein quantitation kit (Bio‐Rad). Next, equal amounts of protein were loaded per sample and separated by 10% SDS‐PAGE electrophoresis before being transferred to a PVDF membrane. The membrane was blocked with 5% milk in TBST with Tween‐20 for 1 h, and then incubated with primary antibodies at 4°C overnight. Specifically, the total OXPHOS Rodent WB Antibody Cocktail (ab110413, Abcam). After several washes, the membrane was incubated with corresponding secondary antibodies for 1 h at room temperature. Finally, protein bands were visualized using an enhanced chemiluminescence detection system (Bio‐Rad), and protein expression levels were calculated by normalizing to Gapdh levels using Image J software.

### 4.12 Follicle counting and Masson's trichrome staining

The ovaries were fixed in 4% paraformaldehyde (PFA) for at least 24 h. After embedding, paraffin wax was used to prepare serial sections of 5 μm thickness. The ovarian serial sections were stained with hematoxylin and eosin (Solarbio). The numbers of primordial, primary, secondary, and atretic follicles were quantified by counting as described previously (Yang, Cong, et al., 2020). To evaluate the extent of ovarian fibrosis in each group, ovarian sections were stained with Masson's Trichrome Stain Kit, following the manufacturer's instructions (Solarbio).

### 4.13 Immunofluorescence staining on ovarian sections

The ovarian sections were blocked with 1% BSA and 0.1% Triton X‐100 in PBS at room temperature for 1 h. The sections were then incubated with QPRT polyclonal antibody (1:100, Proteintech), IDO1 polyclonal antibody (1:100, Proteintech), or PCNA antibody (1:100, Servicebio) overnight at 4°C. After washing the sections several times, Alexa Fluor 488 goat anti‐mouse secondary antibody (Invitrogen) was applied and incubated at room temperature for 1 h in the dark. The sections were then counterstained with 10 μg/mL PI for 10 min and sealed with an anti‐fluorescence quenching agent (Vector) for observation under a laser scanning confocal microscope (Zeiss LSM 700). To assess ovarian cell apoptosis, ovarian sections were stained with TUNEL (Roche) and sealed with an anti‐fluorescence quenching agent. Images were captured using a laser scanning confocal microscope (Zeiss LSM 700).

### 4.14 Transcriptome analysis of ovaries

Total RNA was isolated from mouse ovary using the RNeasy mini kit (Qiagen). The paired‐end libraries were then generated using the TruSeq RNA Sample Preparation Kit (Illumina) following the manufacturer's guide. This involved the purification of poly‐A containing mRNA molecules through the use of poly‐T oligo‐attached magnetic beads. The mRNA fragments were created by fragmentation with divalent cations at 94°C for 8 min. Reverse transcriptase and random primers were then used to generate first strand cDNA from the fragmented RNA. Second strand cDNA synthesis was achieved using DNA Polymerase I and RNase H. The cDNA fragments underwent end repair, the addition of a single ‘A' base, and then adapter ligation. The libraries were purified, enriched via PCR, and quantified using Qubit 2.0 Fluorometer (Life Technologies). The insert size and mole concentration were verified through validation with the Agilent 2100 bioanalyzer (Agilent Technologies). The final libraries were diluted to 10 pM and then sequenced on the Illumina NovaSeq 6000 (Illumina) by Sinotech Genomics Co., Ltd. In the analysis of gene expression, the FPKM values were used to quantify the gene expression levels. The samples from different groups were then subjected to principal component analysis (PCA). The differentially expressed genes (DEGs) were defined as those showing a log2 fold change (FC) greater than 1 and an adjusted *p* < 0.05, calculated using the Benjamini–Hochberg method. The expression pattern of the DEGs was depicted in a heatmap using the pheatmap R package, with the expression of the DEGs related to the mitochondrial respiratory chain complexes displayed in a separate heatmap. The functional implications of the DEGs were determined through KEGG analysis and Gene Set Enrichment Analysis (GSEA), conducted using the clusterProfiler R package.

### 4.15 Statistical analysis

The statistical analysis of the experimental results was performed using the PRISM5 statistical software package. Numerical data were expressed as the mean ± standard error of the mean (SEM). The significance of the differences between the groups was analyzed using independent‐sample t test, One‐Way Analysis of Variance (ANOVA), Tukey's post‐hoc test, or Chi‐square test. *p* < 0.05 was considered statistically significant.

## AUTHOR CONTRIBUTIONS

Qingling Yang conceived the study; Qingling Yang, Hui Li, and Yingpu Sun designed experiments. Hui Li, Huan Wang, Wenhui Chen, Xinxin Zeng, Xiaoyan Luo, Jianmin Xu, and Qingling Yang performed experiments and data collection. H.L. analyzed all data and prepared the figures. Hui Li and Qingling Yang wrote the manuscript.

## CONFLICT OF INTEREST STATEMENT

There is none of the conflict of interest to declare.

## Supporting information


Figure S1.

Figure S2.

Figure S3.

Table S1.

## Data Availability

Data can be obtained from the corresponding author under reasonable request.
