# Supplementary figures and images for: Deletion of enzymes for de novo NAD + biosynthesis accelerated ovarian aging
Source: Aging Cell. 2023 Jun 18;22(9):e13904. doi: 10.1111/acel.13904 (PMC10497836; doi:10.1111/acel.13904)

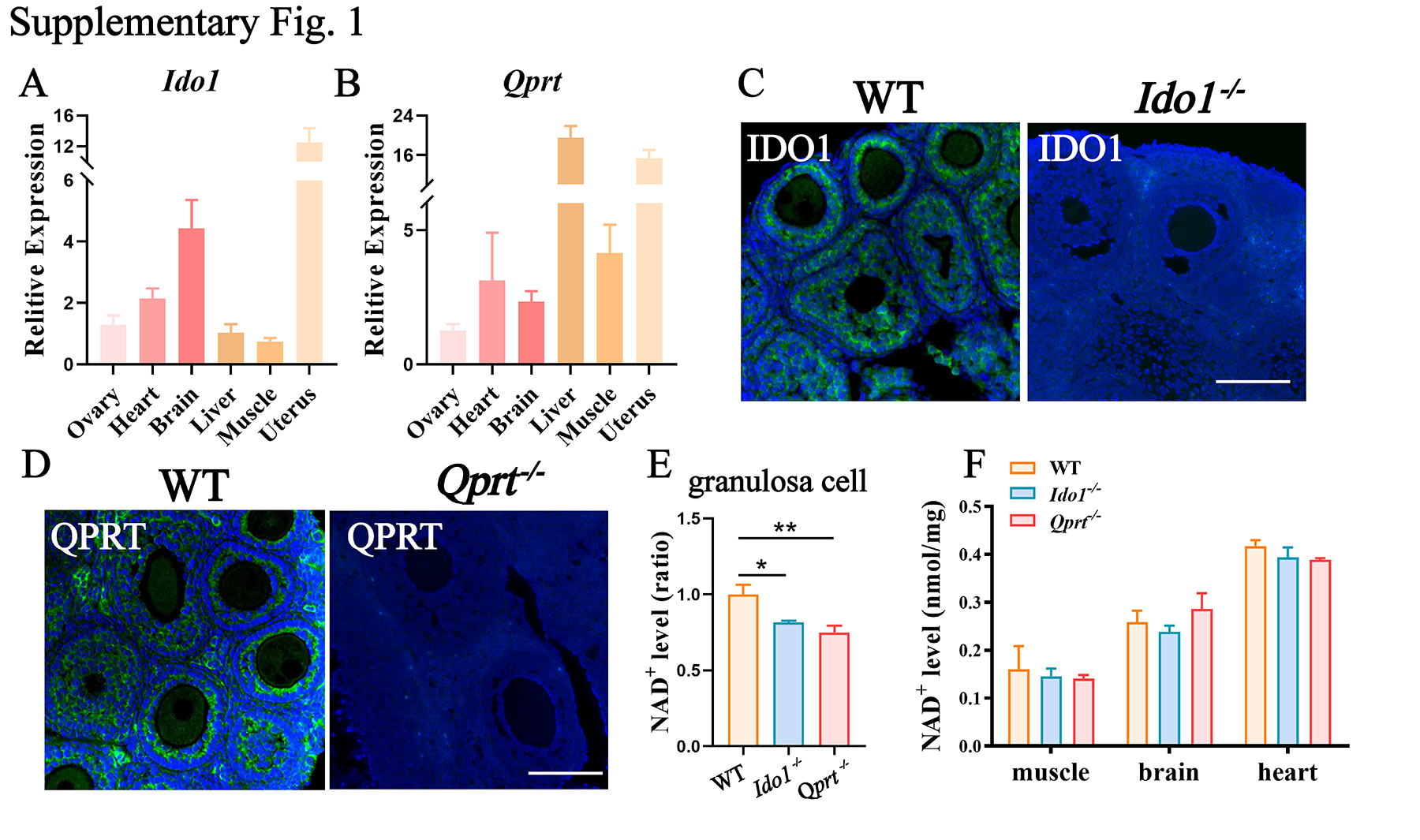

Supplement: Supplementary file 1 — Figure S1. [file ACEL-22-e13904-s002.tif]

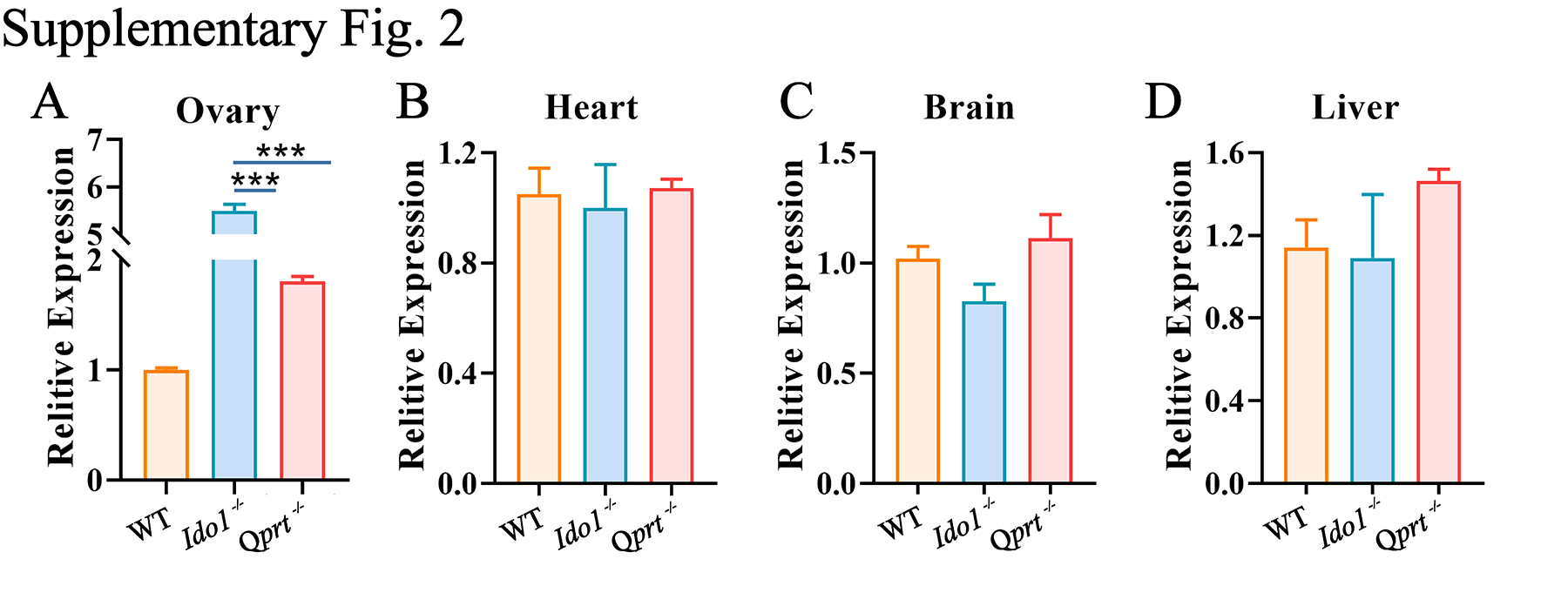

Supplement: Supplementary file 2 — Figure S2. [file ACEL-22-e13904-s003.tif]

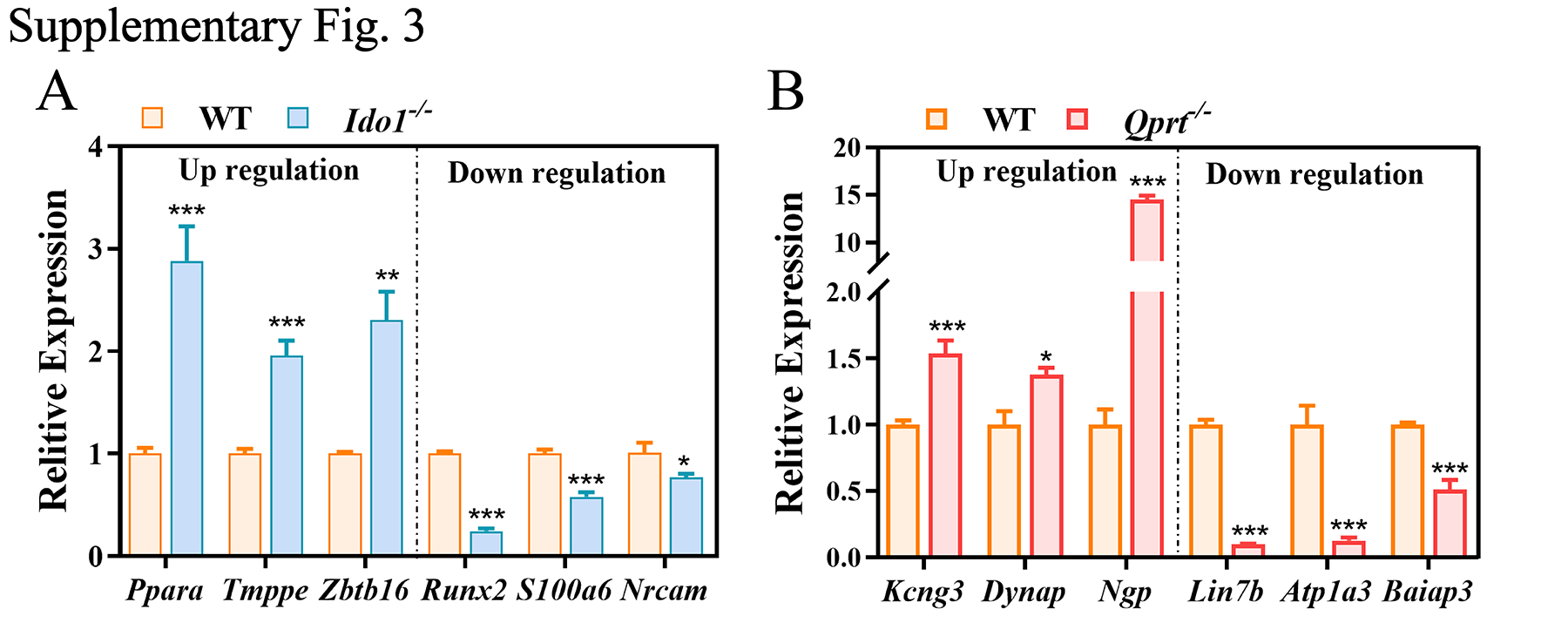

Supplement: Supplementary file 3 — Figure S3. [file ACEL-22-e13904-s001.tif]
