# Supplementary material for: Deletion of enzymes for de novo NAD + biosynthesis accelerated ovarian aging
Source: Aging Cell. 2023 Jun 18;22(9):e13904. doi: 10.1111/acel.13904 (PMC10497836; doi:10.1111/acel.13904)
Supplement: Supplementary file 4 — Table S1. [file ACEL-22-e13904-s004.docx]

**Supplementary figures and figure legends**

**Supplementary Fig. 1 (Related to Fig. 1).**

A-B. Transcript levels of the key enzyme genes *Ido1* (A) and *Qprt* (B) in the ovary, heart, brain, liver, muscle, and uterus detected by real-time RT-PCR from 8-month-old WT mice (n = 3 mice per group).

C. Representative images of immunofluorescence staining of IDO1(green) and nuclei (blue) in WT (left panel) and *Ido1^-/-^* mice (right panel) ovarian sections. Scale bar, 50μm.

D. Representative images of immunofluorescence staining of QPRT (green) and nuclei (blue) in WT (left panel) and *Qprt1^-/-^* mice (right panel) ovarian tissue sections. Scale bar, 50μm.

E. Detection of NAD^+^ levels in WT, *Ido1^-/-^* and *Qprt^-/-^* mice at 8-month-old in granulosa cells isolated from ovaries of WT, *Ido1^-/-^* and *Qprt^-/-^* mice (n = 3 mice for each group).

F. Detection of NAD^+^ levels in WT, *Ido1^-/-^* and *Qprt^-/-^* mice at 8-month-old in muscle, brain, and heart (n = 3 mice for each age).

**Supplementary Fig. 2 (Related to Fig. 2).**

A-D. Transcript levels of the aging-related gene *p21* in the ovary(A), heart(B), brain(C), and liver(D) detected by real-time RT-PCR from 8-month-old WT, *Ido1*^-/-^, and *Qprt*^-/-^ mice (n = 3 mice per group).

**Supplementary Fig. 3 (Related to Fig. 5).**

A and B. Up- and down-regulated genes in the *Ido1^-/-^* (A) and *Qprt^-/-^* (B) mice as compared with controls were verified using RT-PCR.

**Supplementary Table1. List of primer sequences used for real-time RT- PCR analysis**

| Gene | Forward | Reverse |
| --- | --- | --- |
| *Mfn1* | CACTGCAATCTTCGGCCAGTTA | TTTCTGTAGCCCTGTATTTCCACCA |
| *Mfn2* | CTCCAAGTGTCCGCTCCTGAA | AGCTGTCCAGCTCCGTGGTA |
| *Opa1* | GCAGCATTAAGACATGAAATCGAAC | CCAGGGCCTTTGACATTTAGAGA |
| *Fis1* | TGGGCAACTACCGGCTC AA | TTATCAATCAGGCGTTCCAGCTC |
| *Drp1* | GTCCATGAGGAGATGCA GAGG | CACGGGCAACCTTTTAC GA |
| *Ndufv1* | GCGGGTATCTGTGCGTTTCA | GCGCCCATACAGGTTGGTAAAG |
| *Sdhb* | ATCGACACGGACCTCAGCAA | GGTCCTCGATGGACTGCAGATA |
| *Uqcrc2* | AACCCGTGGGATTGAAGCAG | CTGTGGTGACATTGAGCAGGAAC |
| *Atp5a1* | AATCTCCATGCCTCTAACACTCGAC | GCAATACCATCACCAATGCTTAAC |
| *Ppara* | TGCCTTCCCTGTGAACTGAC | CACAGAGCGCTAAGCTGTGA |
| *Tmppe* | TAACGGAGAGACCCCATCGT | CGGGTCAGCTCCGCATAGAT |
| *Zbtb16* | CGTTGGGGGTCAGCTAGAAA | CAGCAGAGGGGACCGGA |
| *Runx2* | GGGAACCAAGAAGGCACAGA | GGATGAGGAATGCGCCCTAA |
| *S100a6* | CCAGACTGCGACACATTCCA | TGGGCTAGAAGAAGCGCAC |
| *Nrcam* | GACGAAGGTAAGAAGGCTGGT | TGGGTCGCGATATCCACTTG |
| *Kcng3* | AGGGAACCCTCCGGGATAAT | TTTGGAGACGATGAACCGCA |
| *Dynap* | AAACGTGGCAGAGCAGTTAT | GGAACACCGGTTCTTCGTCA |
| *Ngp* | AGTGTACTTCCACCCAGGAGA | TGCAATTTCTCTCCTCCCCA |
| *Lin7b* | GCACAGGGCTCAGTGAAACT | TCGAGACTCCAAGGACGTGTA |
| *Atp1a3* | CGCCAAGATGGGGGACAAAA | TCCGGCAGACCTCTTCTACT |
| *Baiap3* | CCAGCTAGTGCGACAGCAA | GCAGCCACCGTGGACA |
| *Col1a1* | GCTCCTCTTAGGGGCCACT | CCACGTCTCACCATTGGGG |
| *Col1a2* | GACACTGGCAACACTGGTAGAGATG | CTCCATCACCACGACTTCCAACAG |
| *Col3a1* | AGTGGGAGGAATGGGTGGCTATC | CTCTCCAGGTCGTCCAGGTCTTC |
| *Gapdh* | AAATGGTGAAGGTCGGTGTGAAC | CAACAATCTCCACTTTGCCACTG |
